# An Unexpected Benefit of the Cashless Society: Cash Use and Endoscopic Oesophageal Foreign Body Removal in England

**DOI:** 10.7759/cureus.117890

**Published:** 2026-10-13

**Authors:** Oliver Sanders, Tarik Abed, Jonathan Philpott

**Affiliations:** 1 Otolaryngology - Head and Neck Surgery, Southend University Hospital, Southend-on-Sea, GBR; 2 Otolaryngology, Southend University Hospital, Southend-on-Sea, GBR

**Keywords:** cashless payments, coin ingestion, hospital episode statistics, oesophageal foreign body, oesophagoscopy, paediatric otolaryngology

## Abstract

Background

Reduced cash use could decrease children’s access to coins and subsequent oesophageal foreign body removal. We examined national trends while distinguishing anatomical coding and endoscopic technique.

Methods

Public Hospital Episode Statistics for England, 2014/15-2024/25, were compared with United Kingdom cash payment shares for 2014-2024. Primary-procedure episodes for flexible and rigid oesophageal removal were combined; children aged 0-14 years and broader upper gastrointestinal coding were examined separately. Correlations of annual levels and first differences were descriptive.

Results

Oesophageal episodes decreased from 1,615 to 1,174 (27.3%), including a reduction from 563 to 330 (41.4%) in children. Cash share decreased from 48% to 9%. Annual-level correlations were strong (all ages, r=0.935; children, r=0.899), whereas first-difference correlations were 0.066 and −0.440. Including broader upper gastrointestinal removal reversed the all-age trend: 3,135 to 3,901 episodes (24.4% increase).

Conclusions

Declining oesophageal removal coincided with declining cash use, but reductions were not proportional. Whilst these aggregate data cannot identify swallowed coins or establish definite causation, they support the hypothesis that an upward trend in cashless payments caused fewer removals of oesophageal foreign bodies.

## Introduction

Coins are common ingested foreign bodies in children, although published object distributions vary by population [1,2]. For United Kingdom (UK) ear, nose, and throat (ENT) surgeons, reduced access to loose change could plausibly reduce urgent oesophagoscopy. Cash’s declining role in payments provides an opportunity to examine this hypothesis [3].

Jangan et al. previously reported declining upper aerodigestive foreign body retrieval after 2012 using Hospital Episode Statistics (HES) through 2021/22 [4]. Notwithstanding periods of incomplete data, this overlapping update extends observation through 2024/25, isolates oesophageal procedures, compares endoscopic coding, and tests whether annual changes accompany changes in cash use.

## Materials and methods

This retrospective ecological study used 11 annual Hospital Episode Statistics admitted-patient workbooks, covering April 2014 to March 2025 [5,6]. All patients represented by included finished consultant episodes (FCEs) in these England tables formed the population. An FCE is a consultant care period, not a unique patient, so repeats may contribute more than once [7]. Reporting followed observational and routinely collected-data guidance [8,9].

The cohort included every FCE with primary G15.1 or G18.1, fibreoptic and rigid oesophageal-removal codes. We summed age bands below 15 years for the paediatric subgroup. Secondary positions, other procedure positions, non-admitted activity, and non-oesophageal codes were excluded; Table 1 sets out codes and roles [10].

**Table 1:** Procedure definitions and analytic roles The Office of Population Censuses and Surveys Classification of Interventions and Procedures, version 4 (OPCS-4), supplies these definitions [10]. G15.1 and G18.1 form the primary outcome; G44.2 is added only for broader coding.

Code	Published procedure description	Analytic role
G15.1	Fibreoptic endoscopic removal of foreign body from oesophagus	Primary outcome
G18.1	Rigid endoscopic removal of foreign body from oesophagus	Primary outcome
G44.2	Fibreoptic endoscopic removal of foreign body from upper gastrointestinal tract	Sensitivity analysis

No sample-size calculation was needed: this was a census of published counts. There were 11 annual observations and 10 annual differences. Age group was the only demographic; sex, object type, comorbidity, speciality, and individual denominators were unavailable.

UK Finance supplied rounded cash-payment shares [3,11-14]. Calendar year t was paired with financial year t/t+1 because nine months overlap. Payment share combines notes and coins and is an indirect exposure measure.

Parameters were annual procedure count, age subgroup, coding, and cash-payment share. We calculated absolute and relative changes and Pearson correlations for annual levels and first differences in Python 3.11 (Python Software Foundation, Wilmington, USA). No p-values, confidence intervals, or alpha threshold were reported: serial dependence and ecological design preclude causal inference.

A sensitivity analysis omitted 2020/21-2021/22, the coronavirus disease 2019 (COVID-19) period, and incomplete 2022/23 data. Only contiguous years contributed differences, and no values were imputed. Public data were accessed in September 2026, without identifiers. 

## Results

All 11 years supplied usable counts. The outcome comprised n=14,497 FCEs, including n=4,875 (33.6%) in ages 0-14 years. Table 2 presents annual counts and cash shares.

**Table 2:** Annual procedure episodes and cash payment shares Counts are primary-procedure finished consultant episodes, expressed as n; cash payment share is the percentage of payments. Endoscopic removal sums fibreoptic and rigid removal, and the final column is the ages 0-14-year subgroup. Cash is assigned to the starting calendar year.

Financial year	Cash payment share (%)	Fibreoptic oesophageal removal (n)	Rigid oesophageal removal (n)	Endoscopic oesophageal removal (n)	Ages 0-14 years (n)
2014/15	48	844	771	1,615	563
2015/16	45	798	733	1,531	523
2016/17	40	787	654	1,441	500
2017/18	34	814	621	1,435	479
2018/19	28	791	562	1,353	430
2019/20	23	764	572	1,336	448
2020/21	17	703	536	1,239	483
2021/22	15	716	483	1,199	405
2022/23	14	580	423	1,003	351
2023/24	12	721	450	1,171	363
2024/25	9	726	448	1,174	330

Oesophageal removal decreased from n=1,615 to n=1,174, a reduction of n=441 (27.3%). In ages 0-14 years, it decreased from n=563 to n=330, a reduction of n=233 (41.4%). The share of cash payments fell from 48% to 9%, a decrease of 39 percentage points.

The lowest all-age count was n=1,003 in 2022/23; it rose by n=171 (17.0%) to n=1,174 in 2024/25, below 2014/15. The paediatric count remained lower at n=330. Figure 1 shows a steadier cash decline and pandemic/reporting-related disturbance in procedures.

**Figure 1:**
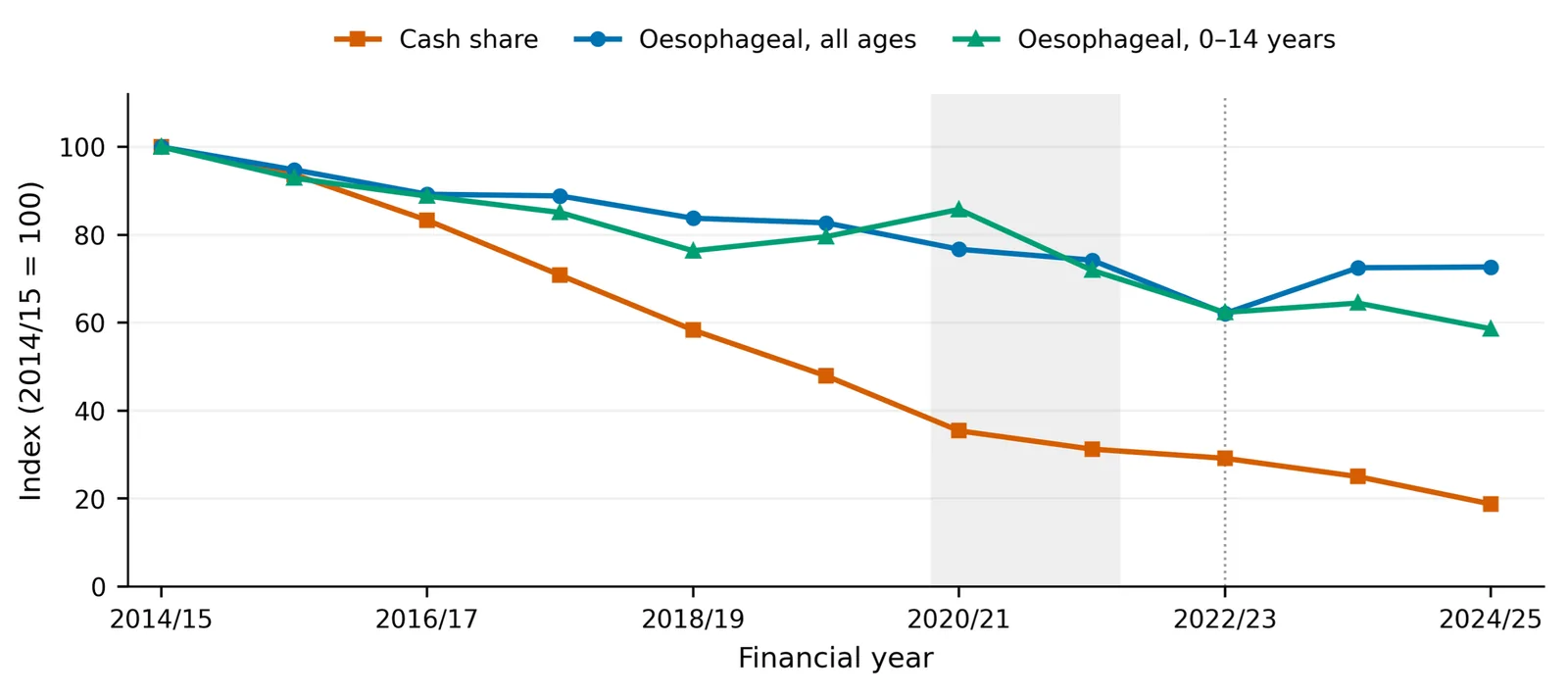
Indexed trends in cash share and oesophageal removal Each series starts at 100. Shading marks 2020/21–2021/22; the dotted line marks incomplete 2022/23 HES reporting. Cash years are aligned as in Table 2. HES: Hospital Episode Statistics

Rigid removal decreased from n=771 to n=448, a reduction of n=323 (41.9%), and fibreoptic removal decreased from n=844 to n=726, a reduction of n=118 (14.0%). Table 3 reports changes and correlations. Annual-level correlations were positive, but first-difference correlations were small or negative.

**Table 3:** Summary change and ecological associations with cash payment share Values are primary-procedure finished consultant episodes, expressed as n; percentage change is relative to 2014/15. r is the Pearson correlation with cash share: levels use 11 pairs, and differences use 10. No p-values or alpha threshold are reported because ecological correlations may be serially dependent; broader upper gastrointestinal episodes additionally include procedure code G44.2 (Fibreoptic endoscopic removal of foreign body from upper gastrointestinal tract - including but not limited to oesophagus).

Episode definition	2014/15 (n)	2024/25 (n)	Absolute change, n (%)	Annual-level r	Annual-difference r
Oesophageal, all ages	1,615	1,174	−441 (−27.3%)	0.935	0.066
Oesophageal, ages 0-14 years	563	330	−233 (−41.4%)	0.899	−0.440
Broader upper gastrointestinal, all ages	3,135	3,901	+766 (+24.4%)	−0.937	−0.242
Broader upper gastrointestinal, ages 0-14 years	680	517	−163 (−24.0%)	0.652	−0.623

With the broader code, all-age counts increased from n=3,135 to n=3,901, an increase of n=766 (24.4%), while paediatric counts fell from n=680 to n=517, a reduction of n=163 (24.0%). Figure 2 shows the increasing all-age G44.2 trajectory. Figure 2 presents the procedure-code trends by age group.

**Figure 2:**
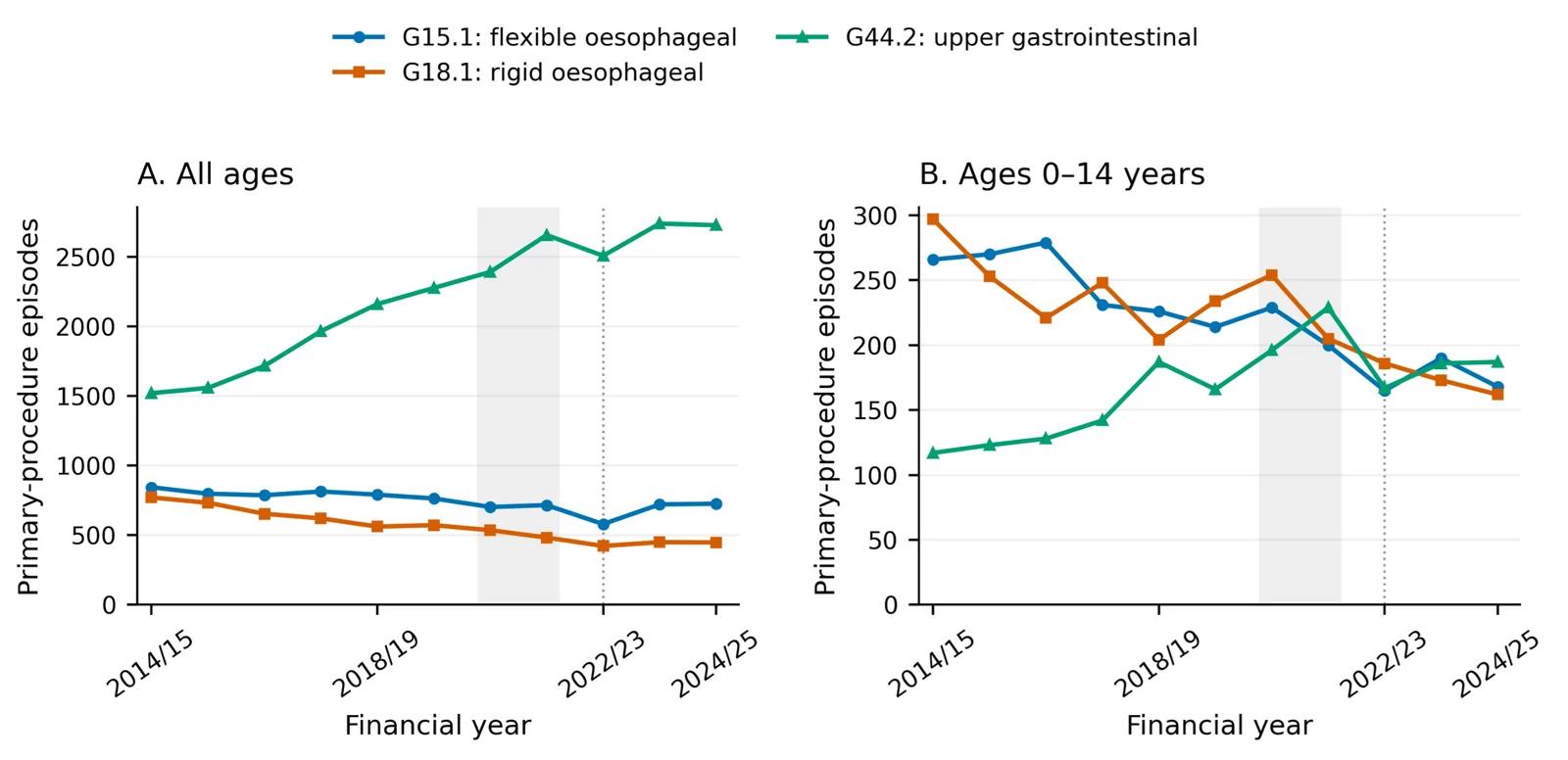
Procedure-code trends by age group Panels show all ages (A) and ages 0-14 years (B). As per Figure 1, shading marks 2020/21–2021/22; the dotted line marks incomplete 2022/23 HES reporting. G44.2 includes anatomical sites beyond the oesophagus. HES: Hospital Episode Statistics

## Discussion

This study extends Jangan et al. by three years and confirms declining oesophageal-coded removal [4]. Their broader upper aerodigestive analysis also declined, while adding G44.2 reversed our all-age trend. A level correlation therefore cannot imply direct annual response because first-difference associations were inconsistent.

The greater paediatric decline is plausible because coins are frequently ingested [1,2]. These data do not identify coins, and payment share is not household coin access. National Audit Office evidence that many issued coins are outside active circulation further weakens one-to-one interpretation [15].

Factors beyond exposure influence procedures. A United Kingdom tertiary-centre study found increased referrals during coronavirus disease 2019 restrictions [16], and presentation, referral, and coding may affect HES activity. Divergent rigid and fibreoptic trends support maintaining both techniques [17]. Adult [18] and paediatric [19] guidance emphasise object-specific management; batteries remain a prevention priority [20].

Limitations are substantial. Hospital Episode Statistics covers England whereas cash estimates are UK-wide; years only approximately align, shares are rounded, and population change was not standardised. Aggregate data cannot distinguish objects or capture sex, comorbidity, speciality, repeat patients, non-admitted care, spontaneous passage or coding accuracy.

Primary-procedure selection omits secondary removal, and G44.2 includes sites beyond the oesophagus. The 2022/23 submission issue and coronavirus disease 2019 period make inference fragile; Pearson correlations cannot control secular confounding or serial dependence. Sensitivity analysis preserved level but not difference correlations, so findings are hypothesis-generating.

## Conclusions

Oesophageal foreign body removal workload declined alongside cash use, particularly in children. The association is compatible with reduced coin exposure but does not establish causation or proportionality. ENT services should retain appropriate retrieval expertise, while future surveillance records the objects removed.
